# Geospatial and multilevel analysis of lifetime HIV testing uptake among high-risk adults in Mozambique: evidence from the 2022–2023 DHS

**DOI:** 10.1186/s12942-026-00462-w

**Published:** 2026-03-26

**Authors:** Thomas kidanemariam Yewodiaw, Helen Lamesgin Endalew, Mihret Getnet, Amare Belete Getahun, Tiget Ayelgn Mengstie, Engidaw Fentahun Enyew, Mequanent Dessie Bitewa, Hiwot Tezera Endale, Tseganesh Asefa

**Affiliations:** 1 Amhara Region Emergency Operation Center, Medical Officer at International Medical Corps, Gondar Field Office, Gondar, Ethiopia; 2 Department of Surgical Nursing, School of Nursing, College of Medicine and Health Sciences, University of Gondar, Gondar, Ethiopia; 3 Department of Human Physiology, School of Medicine, College of Medicine and Health Sciences, University of Gondar, Gondar, Ethiopia; 4 Department of Anesthesia, School of Medicine, College of Medicine and Health Sciences, University of Gondar, Gondar, Ethiopia; 5 Department of Medical Biochemistry, School of Medicine, College of Medicine and Health Sciences, University of Gondar, Gondar, Ethiopia; 6 Department of Human Anatomy, School of Medicine, College of Medicine and Health Sciences, University of Gondar, Gondar, Ethiopia; 7 Department of Public Health, College of Health Sciences, Debre Markos University, Debre Markos, Ethiopia; 8 Department of Reproductive Health, Institute of Public Health, College of Medicine and Health Science, University of Gondar, Gondar, Ethiopia; 9 Armauer Hansen Research Institute, Addis Ababa, Ethiopia; 10 Department of Epidemiology and biostatistics, Institute of public Health, College of Medicine and Health Sciences, University of Gondar, Gondar, Ethiopia

**Keywords:** HIV testing uptake, High-risk adults, Mozambique, Spatial analysis, Multilevel modeling

## Abstract

**Background:**

HIV testing is a critical entry point for prevention and treatment, yet disparities in uptake persist among high-risk populations in Mozambique. This study investigates the prevalence, spatial distribution, and determinants of lifetime HIV testing among high-risk adults aged 15–49 using 2022–2023 DHS data.

**Methods:**

A cross-sectional analysis of 15,393 high-risk adults was conducted. Descriptive statistics, spatial analyses (Moran’s I, hotspot analysis, Kriging interpolation, SaTScan), and multilevel logistic regression models were applied to identify individual- and community-level factors associated with HIV testing uptake. We estimated the weighted prevalence of lifetime HIV testing uptake. Adjusted odds ratios (AORs) with 95% confidence intervals were reported to measure the association between explanatory variables and HIV testing uptake, while statistical significance was determined using p-values < 0.05.

**Results:**

Overall, 63.7% of high-risk adults had ever tested for HIV. Testing uptake was higher among females (66%) than males (57%), and among urban (76%) versus rural residents (56%). The highest testing rate was in the 25–34 age group (78%) and lowest among adolescents 15–19 years (31%). Wealth, education, marital status, employment, media exposure, and HIV knowledge positively influenced testing. Significant regional disparities were observed, with southern provinces (Maputo City 87.7%) showing higher uptake than northern and central provinces (Zambezia 44.9%). Spatial analysis confirmed strong clustering (Moran’s I = 0.78, *p* < 0.001), identifying low-testing hotspots in northern and central rural areas. Multilevel modeling showed individual and community factors explained 62.9% of between-cluster variance, with females (AOR = 2.39), higher education (AOR = 6.44), marriage (AOR = 4), and urban residence (AOR = 1.58) significantly increasing odds of testing.

**Conclusion:**

HIV testing uptake in Mozambique remains uneven across socio-demographic and geographic groups. Targeted and equity-focused interventions are needed to expand testing among adolescents, men, rural populations, and residents of northern and central provinces. Strengthening community-based testing services, improving health education, and addressing geographic barriers will be essential for accelerating progress toward national HIV control targets

## Introduction

HIV remains a significant public health issue in Sub-Saharan Africa, with significant gaps in testing coverage and treatment access, especially among key populations and underserved regions [1]. Mozambique continues to face a significant HIV epidemic, with an estimated adult HIV prevalence of 8.1% [2]. Despite increased efforts to expand HIV testing services, uptake of HIV testing remains unequal across geographic regions and population categories, limiting timely diagnosis and treatment commencement to achieve UNAIDS 95-95-95 targets, hindering timely initiation of antiretroviral therapy and reducing transmission [1]. Understanding the spatial characteristics of both HIV testing uptake and HIV status (positive or negative) is crucial for informing targeted treatments [3]. HIV testing uptake represents a critical doorway to HIV care and prevention; yet, discrepancies persist by age, gender, residency, and area, resulting in pockets of persons with poor testing coverage and unknown HIV status [1, 4, 5]. Likewise, HIV prevalence and test positivity rates vary geographically, frequently clustering in hotspots connected to social, economic, and health service determinants [2]. Regional comparisons reveal significant disparities in testing uptake across East and West Africa, with Eastern countries like Kenya and Tanzania showing improvements. In contrast, West African countries like Nigeria and Mali face low HIV test uptake [2, 6, 7]. UNAIDS and PEPFAR’s strategies emphasize reaching unreached populations, particularly adolescent girls and young women, through community-based and mobile testing, and The Global Fund’s 2023–2028 Strategy aims to provide data-driven interventions in high-burden Sub-Saharan Africa [1].

Despite ongoing HIV prevention efforts, disparities remain among people with disabilities, individuals aged 15–24 years [2, 8], and those with low education. Combining spatial analytic methods with multilevel modeling provides robust tools to highlight regional disparities and community-level implications on HIV testing [6]. National estimates provide an overview, but localized evidence is needed to identify areas with low testing coverage [1]. This is especially significant for Mozambique, where regional disparities in epidemic intensity and service access are prominent [2].

HIV testing uptake in sub-Saharan Africa remains uneven, with lower rates among adolescents, men, rural residents, and those with less education or income [9]. In Mozambique, testing behaviors vary markedly across regions, shaped by health service access, education, and socio-cultural norms [10]. Spatial epidemiological methods, such as Moran’s I, hotspot analysis, Kriging interpolation, and SaTScan, reveal geographic clustering and identify underserved “Hotspots,” enabling targeted resource allocation [11]. Integrating these approaches with multilevel modeling enhances understanding of both spatial and socio-demographic disparities in HIV testing.

Although previous studies in Mozambique have examined socio-demographic determinants of HIV testing, few have integrated spatial analysis with multilevel modeling to simultaneously assess individual and contextual factors. The Demographic and Health Survey (DHS) offers a unique opportunity to address this gap, as it provides nationally representative data with rich individual- and community-level variables, along with geocoded cluster information.

This study aimed to: [1] assess the prevalence of lifetime HIV testing among high-risk adults in Mozambique; [2] examine spatial patterns and clusters of testing uptake; and [3] identify individual- and community-level determinants of testing using multilevel modeling. Findings from this research can inform tailored, equity-focused strategies to improve testing coverage, particularly in underserved populations and regions, thereby advancing progress toward national and global HIV control targets.

## Methodology

### Study design and data source

This study used a cross-sectional design based on data from the 2022–2023 Mozambique Demographic and Health Survey [2]. The DHS is a nationally representative survey that collects data on a wide range of health indicators using standardized questionnaires [12]. We specifically used the individual recode datasets (IR for women and MR for men) available through the DHS Program after obtaining permission [8].

The survey employed a two-stage stratified sampling technique, stratified by urban and rural areas within each province [2]. This analysis focuses on populations represented in the DHS dataset, as key populations and individuals with disabilities are not directly captured.

### Conceptual framework and theoretical basis

**Fig. 1:**
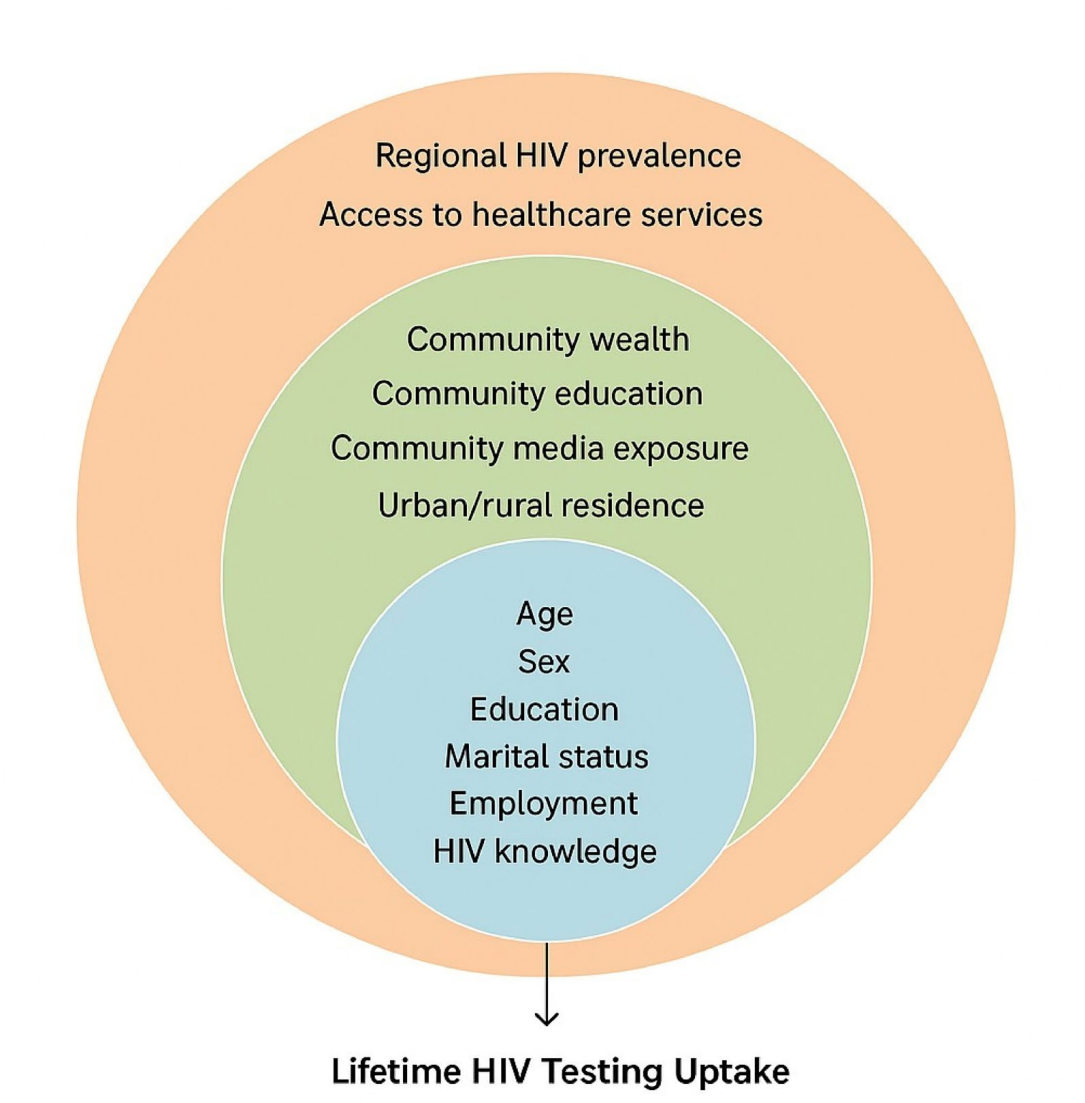
Conceptual framework illustrating individual, interpersonal, community, and structural determinants of lifetime HIV testing uptake among high-risk adults in Mozambique

This study was guided by the Socio-Ecological Model (SEM), which emphasizes that health behaviors, such as HIV testing, are shaped by factors operating at multiple, interacting levels. At the individual level, demographic and behavioral factors including age, sex, education, risk behavior, and media exposure may influence testing decisions. Interpersonal factors, such as marital status, partner communication, and household composition, can affect awareness and motivation to test. At the community level, contextual characteristics like aggregated education, wealth, urbanization, and region shape local norms, service access, and infrastructure [13–20]. By applying SEM, the study captures the complex interplay between individual- and contextual-level determinants of lifetime HIV testing uptake among high-risk adults in Mozambique. This framework also guided the selection and categorization of variables and supports the use of multilevel modeling to appropriately account for the nested nature of these influences (Fig. 1).

### Study population

The target population includes high-risk persons aged 15–49 years, determined based on reported behaviors or situations that increase HIV vulnerability [21]. High-risk individuals include those with multiple sexual partners in the past 12 months, STI history, transactional sex, or inconsistent condom use with non-marital partners [18]. The male and female DHS datasets were initially analyzed separately to account for sex-specific variables and then harmonized by aligning common variables before merging for combined analyses where necessary [22].

### Outcome variable

The primary outcome was lifetime HIV testing uptake, defined as whether an individual had ever been tested for HIV, regardless of the result. This was measured as a binary variable: 1 = Yes (ever tested) and 0 = No (never tested), based on self-report [8, 15]. HIV testing was assessed for individuals in the DHS questionnaire, and the question framing regarding recent risky sexual behavior was verified for accurate interpretation.

### Operational definitions

High-risk adults are individuals aged 15–49 who report one or more behaviors that increase their vulnerability to HIV acquisition, including recent sexual activity [23], multiple sexual partnerships, history of sexually transmitted infections (STIs), and lack of condom use during last sexual encounter [24].

HIV knowledge in this study is defined as a correct understanding of key facts about HIV transmission and prevention. It is measured using five DHS questions on common misconceptions (e.g., transmission by mosquitoes or sharing food), awareness that healthy-looking individuals can have HIV, and knowledge of prevention methods like consistent condom use and having one uninfected faithful partner. Each correct answer scores one point, creating a composite score from 0 to 5. The score is categorized as low (0–2), moderate [3], and high ([4, 5]), or dichotomized into good (≥ 4) versus poor (< 4) knowledge. This classification follows standard DHS methods to assess HIV awareness linked to prevention behaviors [25].

Comprehensive HIV Knowledge: A composite score based on correct responses to five standard DHS questions about HIV transmission and prevention, categorized as poor or good knowledge (see previous HIV knowledge definition).

Risky sexual behavior is defined as engaging in one or more behaviors that increase HIV risk, such as multiple sexual partners, inconsistent condom use with non-regular partners, sex with casual or commercial partners, or sex under the influence of alcohol or drugs. Coded as binary (1 = Yes, 0 = No) [26–28].

Health Care Decision-Making Autonomy: Women who make health care decisions alone or jointly with their partner are coded as autonomous (1, while those whose health decisions are made solely by others are coded non-autonomous (0) [26, 29].

Exposure to Media: Frequency of accessing media sources (radio, TV, newspapers) at least once a week, coded as exposed [1] or not exposed (0) [2].

Wealth Index: Composite index based on household assets and amenities, categorized into quintiles from lowest to highest wealth [30].

### Community-level variables

Community-level variables were generated by aggregating individual-level data within clusters (primary sampling units) to capture the broader contextual influences on HIV testing uptake. The following community-level factors were included: Community-level wealth: calculated as the mean household wealth index within each cluster [31, 32]. Community-level education: measured as the proportion of adults in the cluster with at least secondary education [32]. Community-level media exposure: defined as the proportion of individuals in the cluster who reported regular access to media (TV, radio, or newspapers) [33, 34].

### Independent variables

The study identifies various factors affecting HIV lifetime testing, including age, sex, education level, marital status, wealth index, employment status, media exposure, comprehensive HIV knowledge, risky sexual behavior, and community-level factors [13–20]. The multilevel model included individual-level variables and contextual factors at the community or regional level. Beyond urban/rural residence, additional contextual determinants such as regional HIV prevalence, healthcare access, and media exposure were considered or discussed to account for potential regional influences on HIV testing uptake.

### Sample size determination and procedure

This study used data from the 2022–2023 Mozambique Demographic and Health Survey (MDHS), which applied a two-stage stratified cluster sampling technique to ensure national representativeness. A total of 619 clusters (198 urban and 414 rural) were selected, followed by systematic sampling of 28 households per cluster. Eligible individuals included women aged 15–49, men, and women residing in selected households.

For this analysis, the sample focused on high-risk adults, identified based on behavioral and demographic risk factors. After excluding records with missing or incomplete HIV testing information, the final weighted sample included 15,393 individuals (4,272 men and 11,121 women) across 616 georeferenced clusters. This sample was used for both multilevel and geospatial analyses to assess the spatial distribution and determinants of lifetime HIV testing uptake. All analyses were conducted using survey weights and procedures that account for the complex sampling design, which also appropriately handle missing data without the need to exclude observations.

### Data management and weighting

Data were cleaned, recoded, and merged using Stata version 17 [35].

The svyset command was used to ensure national representativeness and valid standard errors in sampling weights, clustering, and stratification [22]. For spatial analysis, GPS coordinate data were merged from the DHS spatial datasets [36]. All analyses accounted for the complex survey design of the DHS, including stratification, clustering, and unequal probabilities of selection. Sampling weights provided by the DHS were applied to produce nationally representative estimates. For the subset of high-risk adults, weights were normalized within the subset to maintain correct representation without inflating population totals. Weighted multilevel logistic regression models and spatial analyses (Kriging and SaTScan) incorporated these adjustments, ensuring unbiased estimation of prevalence, cluster-level proportions, and determinants of HIV testing uptake while properly accounting for clustering and stratification.

### Statistical analysis

#### Descriptive and bivariate analysis

The distribution of lifetime HIV testing uptake across explanatory variables was calculated using frequencies and weighted proportions. The study used Chi-square tests to analyze the distribution of lifetime HIV testing uptake across explanatory variables [37].

### Multilevel logistic regression

The hierarchical structure of the Demographic and Health Survey (DHS) data two-level mixed-effects logistic regression model (random intercept at the cluster level) was used. All independent variables were included in the model, with clusters treated as random effects to account for within-cluster correlation [38]. Four models were constructed to assess community-level variance, including empty, adjusted for individual and community-level variables, and full models including both factors [39]. The study utilized intra-class correlation coefficient (ICC), Median Odds Ratio (MOR), and Proportional Change in Variance (PCV) to evaluate community-level variation [40]. Model fitness was compared using log-likelihood, AIC, and BIC values [41]. Variables with *p* < 0.05 in the final model were considered statistically significant.

### Spatial analysis

Spatial analysis was conducted using ArcGIS Pro 10.7, SaTScan, and Stata 17. The objectives were to assess the geographic distribution of HIV testing uptake and identify hotspot areas. Global Moran’s I spatial autocorrelation analysis was used to assess whether HIV testing uptake were spatially clustered, dispersed, or randomly distributed across Mozambique [42]. Hotspot analysis using Getis-Ord Gi* This method identified geographic hotspots and cold spots for low and high HIV testing uptake at the cluster level, respectively [43]. Spatial interpolation was performed using ordinary kriging based on 620 sampled clusters. Predicted HIV testing prevalence was estimated at unsampled locations using the semi-variogram model fitted to observed cluster data. Kulldorff’s spatial scan statistic was used to detect significant spatial clusters of low and high HIV testing uptake using a Bernoulli model [5]. Confidence intervals (CIs) or prediction bands were reported for spatial predictions and modeled estimates to convey the uncertainty of the findings.

### Ethical considerations

This study is a secondary analysis of the 2022–2023 Mozambique Demographic and Health Survey (DHS) data, publicly available from the DHS Program (https://dhsprogram.com). Access to the dataset was granted in March 2024 for a previously approved research project, before the temporary suspension of the DHS data portal. Ethical approval for the original survey was obtained by the implementing agencies from the Mozambique National Committee on Bioethics for Health (CNBS) and the Institutional Review Board (IRB) of ICF International, in accordance with U.S. Department of Health and Human Services regulations. All DHS datasets are anonymized before release to protect participant confidentiality. As the present study analyzed de-identified secondary data, no additional ethical approval was required. All analyses complied with the DHS Program’s data use policy [2]. Direct link to the dataset listing: https://dhsprogram.com/data/available-datasets.cfm. All GPS data were displaced as per DHS guidelines. No sensitive location information is reported, and cluster coordinates are presented at an aggregated level to maintain confidentiality.

## Results

### Descriptive statistics

Among 15,393 high-risk adults in Mozambique’s 2022–2023 DHS, 72% were female, with females showing higher HIV testing uptake (66%) than males (57%). Testing was most common in the 25–34 age group (78%) and lowest among adolescents 15–19 years (31%). HIV testing increased with education level, from 51% among those with no education to 76% for secondary or higher. Testing uptake also rose with wealth, ranging from 49% in the poorest to 80% in the richest quintile. Urban residents had substantially higher uptake (76%) compared to rural residents (56%). Formerly married and currently married adults had higher testing rates (75% and 70%, respectively) than never-married individuals (34%). Employment and media exposure were associated with higher testing 71% among the employed and 73% among those regularly exposed to media versus 58% and 57% among their counterparts. Finally, hearing about HIV was linked to increased testing (70% vs. 60%) (Table 1).

**Table 1:**
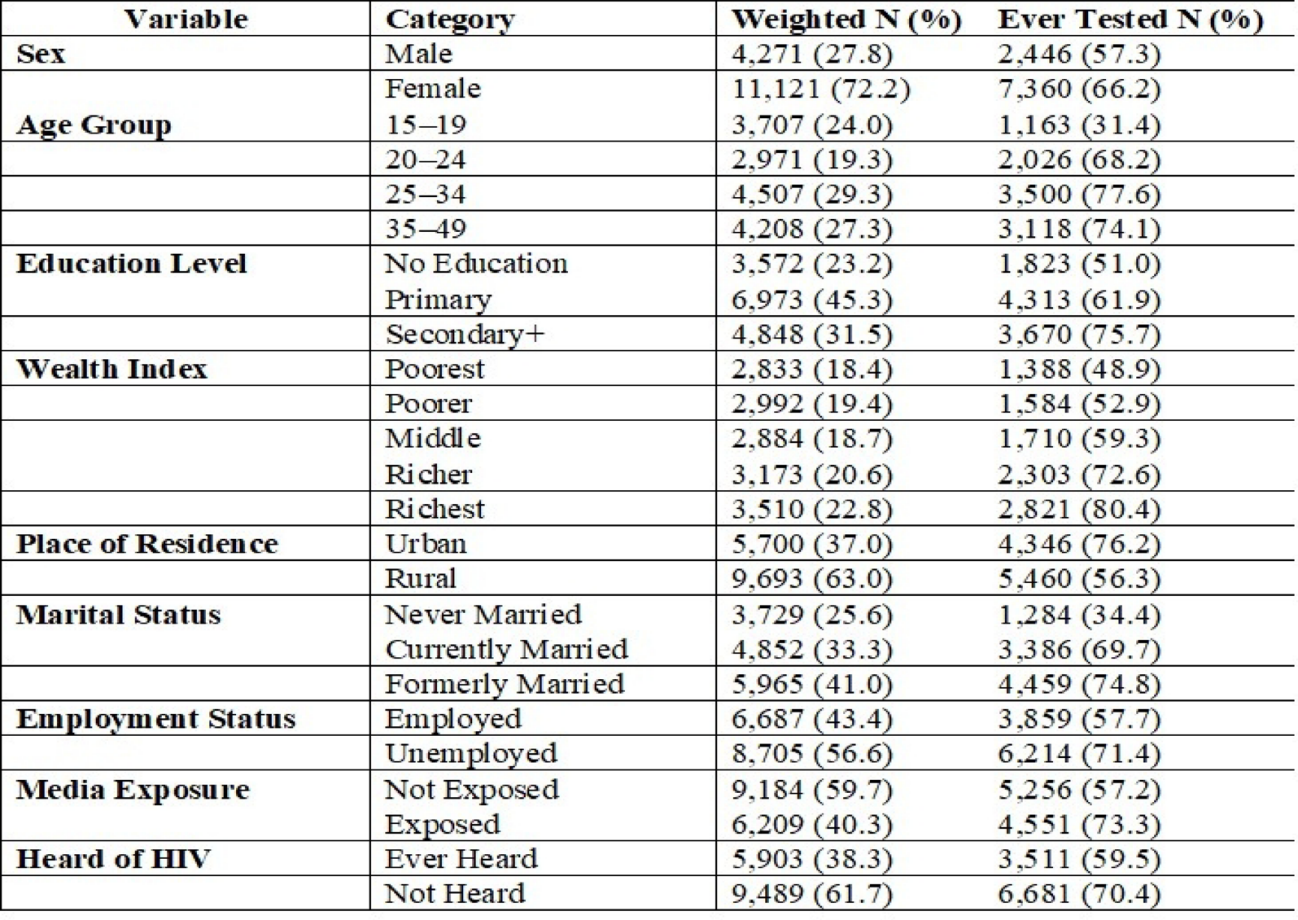
Socio-demographic Characteristics and Lifetime HIV Testing Uptake among High-Risk Adults in Mozambique, DHS 2022–2023 (N = 15,392)

### Proportions lifetime HIV testing by region and residence

**Fig. 2:**
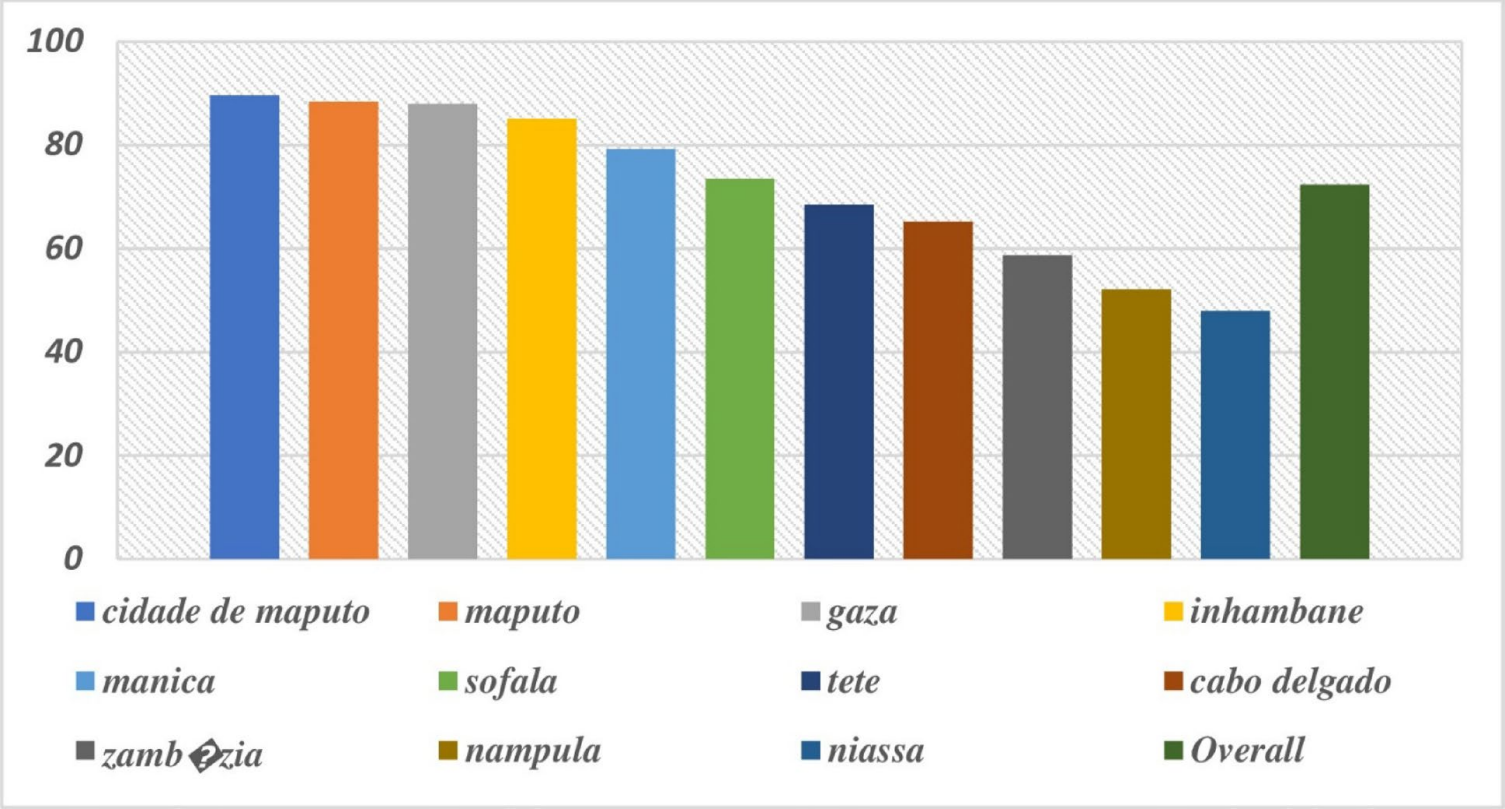
Proportion of High-Risk Adults Ever Tested for HIV by Province in Mozambique, 2022–2023

A total of 15,393 high-risk adults aged 15–49 was included in the analysis. Among them, 63.7% (95% CI: 62.9%–64.5%) reported having ever tested for HIV, while 36.3% (95% CI: 35.5%–37.1%) had never undergone testing. Lifetime HIV testing uptake in Mozambique varies by region, highest in Cidade de Maputo (87.7%), Maputo (86.3%), and Gaza (84.2%), moderate in Niassa (48.9%), Nampula (48.0%), and Zambézia (44.9%), and lowest in Inhambane (21.3%) and Manica (25.2%) (Fig. 1). HIV testing uptake was higher in urban areas (76.2%) than rural areas (56.3%), and this difference was statistically significant (χ² = 45.7, *p* < 0.001), indicating clear regional disparities (Table 1; Fig. 2).

### Spatial autocorrelation

**Fig. 3:**
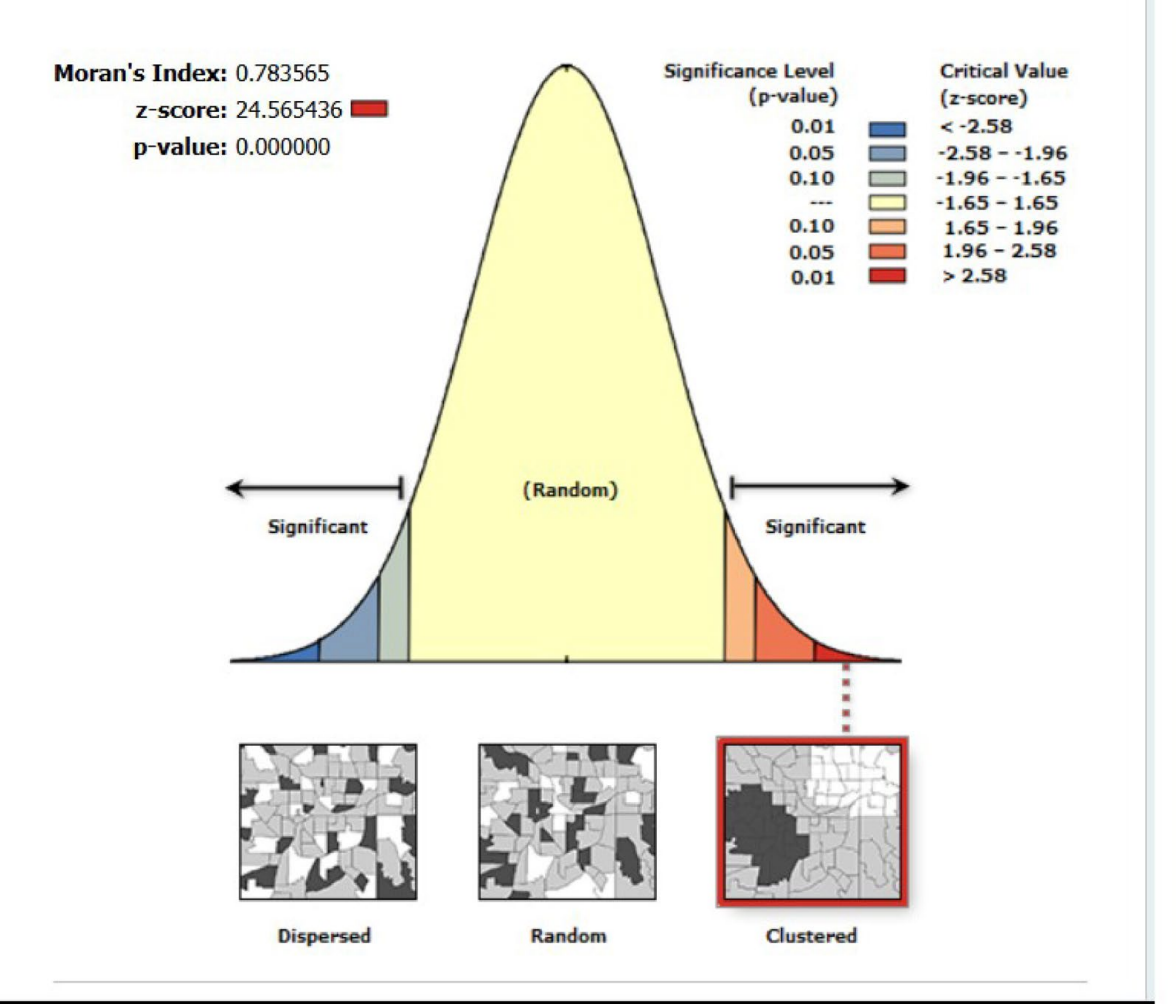
Spatial autocorrelation analysis of HIV testing uptake among high-risk adults in Mozambique, 2022/2023 MDHS

Moran’s I (0.78) shows strong clustering of similar HIV testing rates. The significant z-score (24.57) and p-value (< 0.001) confirm that this pattern is unlikely to be due to chance, indicating that neighboring areas have similar testing uptake **(**Fig. 3).

### Hot and cold spot analysis

The hotspot analysis of HIV testing uptake in Mozambique (DHS 2022/2023) reveals clear regional disparities. Low HIV testing uptake (hot spots) is concentrated in the northern and central provinces, particularly in Zambezia, Niassa, Cabo Delgado, and Tete, where rural and hard-to-reach communities dominate. In contrast, higher HIV testing uptake (cold spots) is observed in southern and urban areas, including Maputo City, Maputo Province, Gaza, and Inhambane, as well as parts of Beira and Nampula City (Fig. 3).

**Fig. 4:**
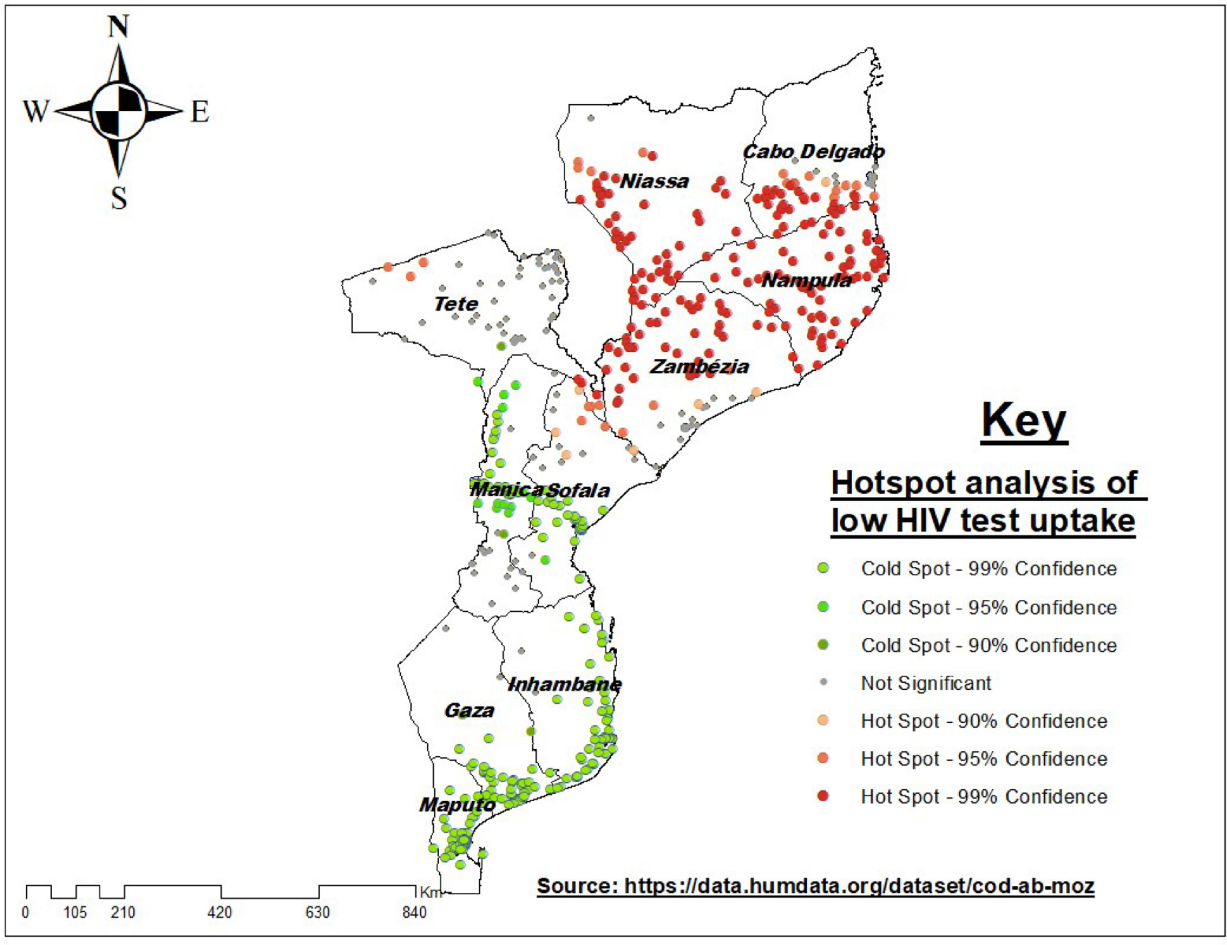
Hotspot and cold spot analysis of low proportion HIV testing uptake among high-risk adults in Mozambique, 2022/2023 MDHS

### Interpolation (Ordinary kriging)

**Fig. 5:**
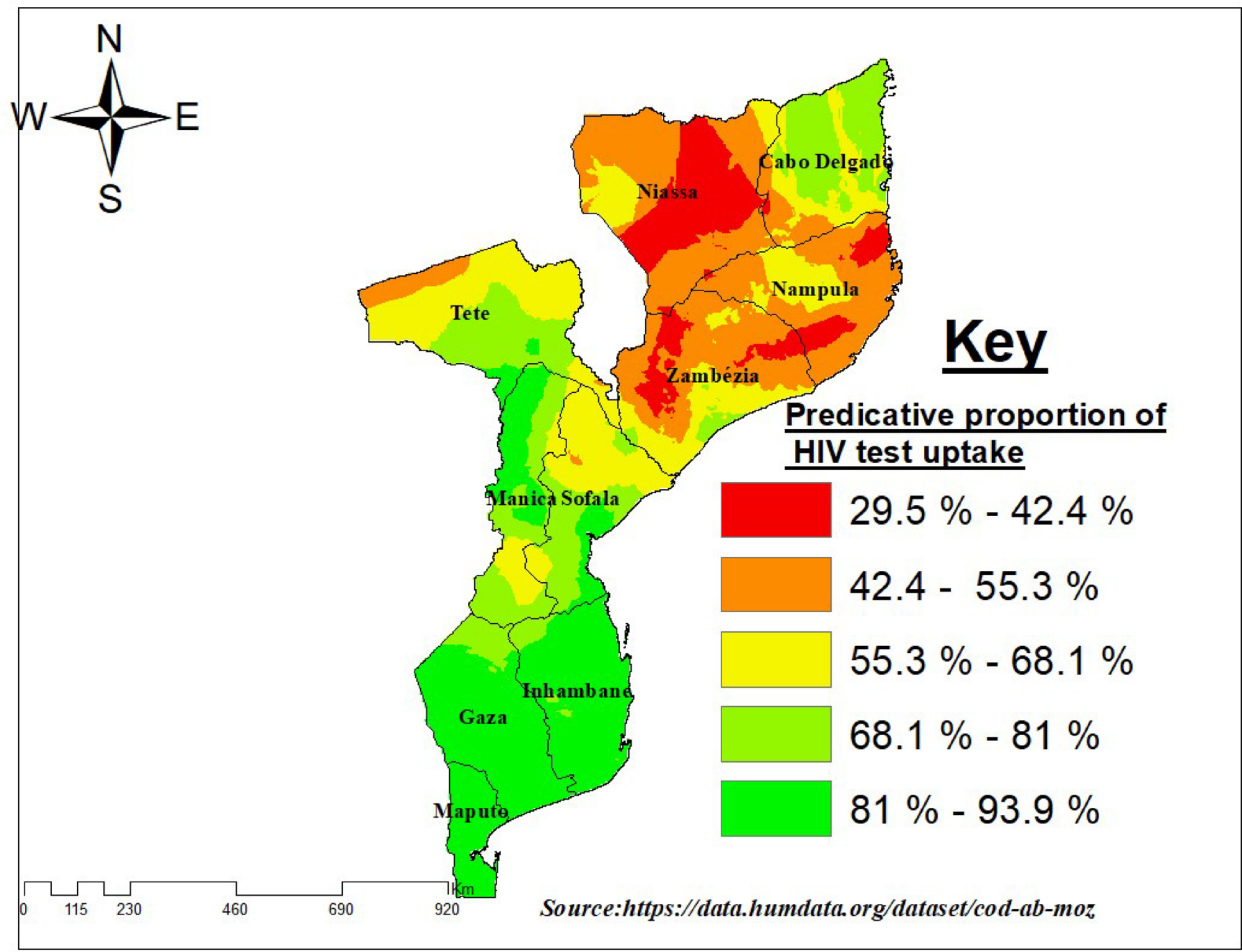
Spatial distribution of lifetime HIV testing uptake among high-risk adults in Mozambique, 2022–2023 DHS

The spatial interpolation map reveals clear geographic disparities in lifetime HIV testing uptake across Mozambique. Higher predicted uptake is concentrated in southern regions, such as Maputo City, Maputo Province, and Gaza, likely reflecting better access to health services, higher urbanization rates, and stronger awareness campaigns. These areas are visualized in cooler colors on the map. Conversely, central and northern regions, including Zambezia, Nampula, and Cabo Delgado, show lower predicted uptake, depicted in green color with brighter colors on the map. To illustrate the spatial distribution of lifetime HIV testing uptake across Mozambique, we generated an interpolated surface map (Fig. 4). Although a point-based map of DHS clusters was initially considered, the overlapping points and lack of spatial coverage representation limited its interpretability. The interpolated surface provides a more comprehensive visualization by estimating values between sampled clusters, thereby highlighting geographic variations and potential hotspots with greater clarity. This approach has been widely applied in previous DHS-based spatial studies and offers a clearer depiction of areas with relatively higher or lower HIV testing uptake (Fig. 5).

### Spatial distribution of low HIV testing uptake

The spatial scan statistics identified clear geographic variations in HIV testing uptake across Mozambique. A large and statistically significant primary cluster of low testing uptake was detected in the northern region, covering Niassa, Cabo Delgado, Nampula, and northern Zambézia. This primary cluster had a relative risk (RR) of 2.65 (*p* < 0.001), indicating that individuals residing within this zone were more than twice as likely to exhibit low HIV testing uptake compared with those living outside the cluster. The primary cluster encompassed over 260 DHS enumeration areas and covered a wide geographic radius of approximately 756 km, suggesting a broad region of concentrated vulnerability.

**Fig. 6:**
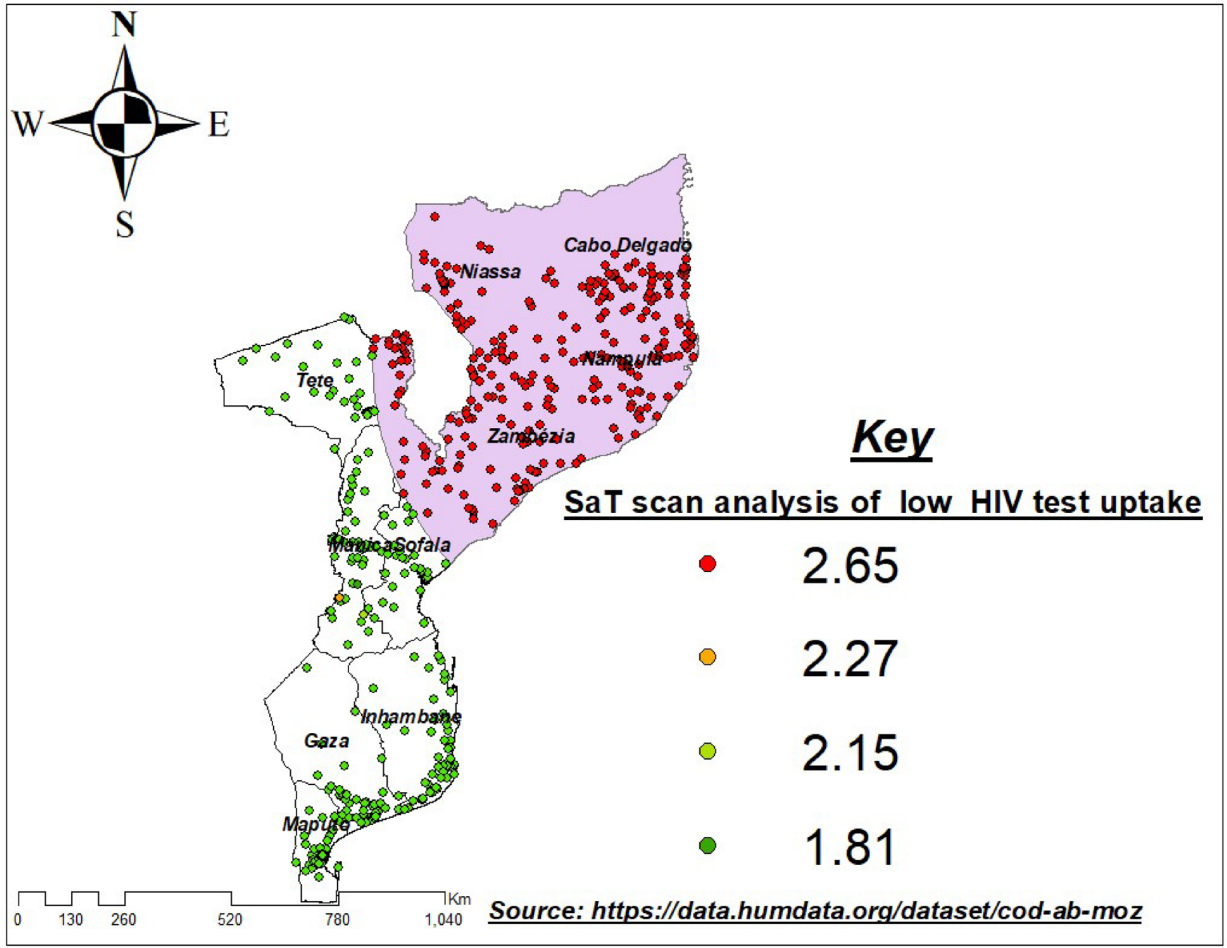
SaTScan-Identified Significant Spatial Clusters of Low HIV Testing Uptake among high-risk adults in Mozambique, 2022/2023 MDHS

A secondary, localized cluster was detected in central Mozambique, centered on the Sofala–Manica area. This cluster had an RR of 2.15 (*p* = 0.0046) and included three DHS clusters. Although smaller in size, this cluster remained statistically significant and represented a distinct pocket of elevated risk for low HIV testing uptake. ArcGIS mapping confirmed these statistical findings by visually highlighting dense concentrations of high-risk clusters in the Northern provinces (shown in red), moderate-risk clusters in central areas (yellow), and predominantly low-risk clusters across the southern provinces of Maputo, Gaza, and Inhambane (green). Overall, the results demonstrate a strong north–south geographic gradient in HIV testing uptake in Mozambique. The northern provinces exhibited widespread low uptake, central provinces showed isolated pockets of low testing behavior, and the southern region showed consistently higher testing uptake (Table 2: Fig. 6):

**Table 2:**
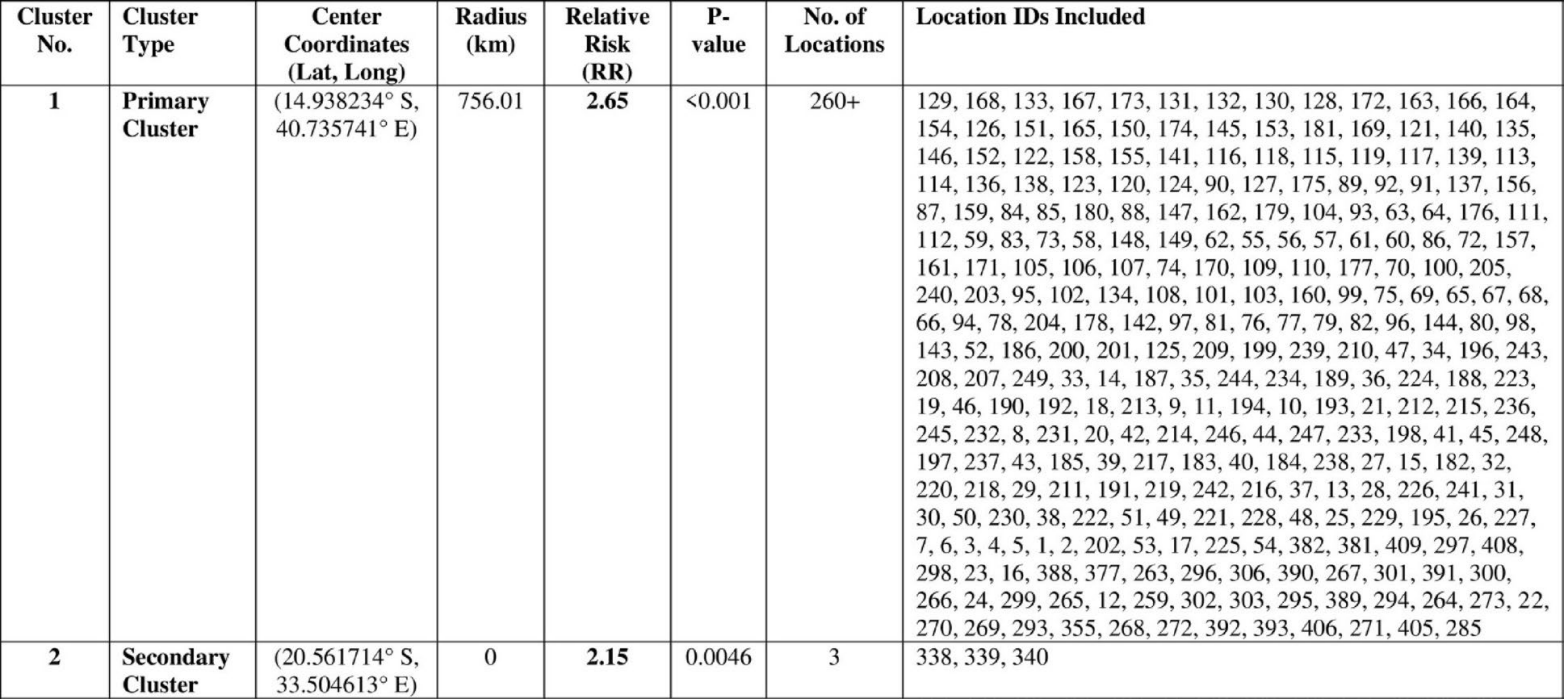
Spatial Distribution of Primary and Secondary SaTScan Clusters of Low HIV Testing Uptake among high-risk adults in Mozambique, 2022/2023 MDHS

#### Multilevel logistic regression analysis

The multilevel logistic regression analysis revealed significant individual and community-level factors influencing HIV testing uptake among high-risk adults in Mozambique. In the fully adjusted model (Model 3), females had significantly higher odds of HIV testing compared to males (AOR: 2.39; 95% CI: 2.11–2.71). Older age groups were more likely to be tested, especially those aged 25–34 (AOR: 4.45; 95% CI: 3.78–5.24). Education showed a strong positive gradient; individuals with secondary (AOR: 3.43; 95% CI: 2.89–4.07) and higher education (AOR: 6.44; 95% CI: 4.01–10.34) were significantly more likely to undergo HIV testing compared to those with no education. Being currently or previously married was associated with nearly fourfold higher odds of testing. Employment (AOR: 1.55; 95% CI: 1.37–1.74), media exposure (AOR: 1.17; 95% CI: 1.04–1.31), higher wealth status (rich: AOR: 1.30; 95% CI: 1.07–1.57), moderate HIV knowledge (AOR: 1.55; 95% CI: 1.35–1.78), and engaging in risky sexual behavior (AOR: 7.71; 95% CI: 6.34–9.36) were all positively associated with HIV testing.

At the community level, rural residents were less likely to be tested (AOR: 0.63; 95% CI: 0.51–0.78) compared to urban counterparts. Some regional differences were substantial; for instance, compared to Niassa, higher odds of HIV testing were observed in Inhambane (AOR: 5.19; 95% CI: 3.73–7.23), Manica (AOR: 4.14; 95% CI: 3.06–5.60), Sofala (AOR: 2.26; 95% CI: 1.70–3.01), Maputo Province (AOR: 7.53; 95% CI: 5.27–10.76), and Maputo City (AOR: 6.98; 95% CI: 4.78–10.19).

Model fit improved progressively, with Model 3 demonstrating the best fit based on the lowest AIC (12,485.57) and BIC (12,729.33), along with a reduced cluster variance (σ² = 0.2862). The intraclass correlation coefficient (ICC) decreased from 19% in the null model to 8% in the final model, suggesting that individual and community-level variables accounted for a considerable portion of the between-cluster variability. The proportional change in variance (PV) was 62.94% in Model 3, and the median odds ratio (MOR) declined from 2.11 in the null model to 1.67, further indicating that contextual variation in HIV testing was reduced after adjusting for covariates (Table 3).

**Table 3:**
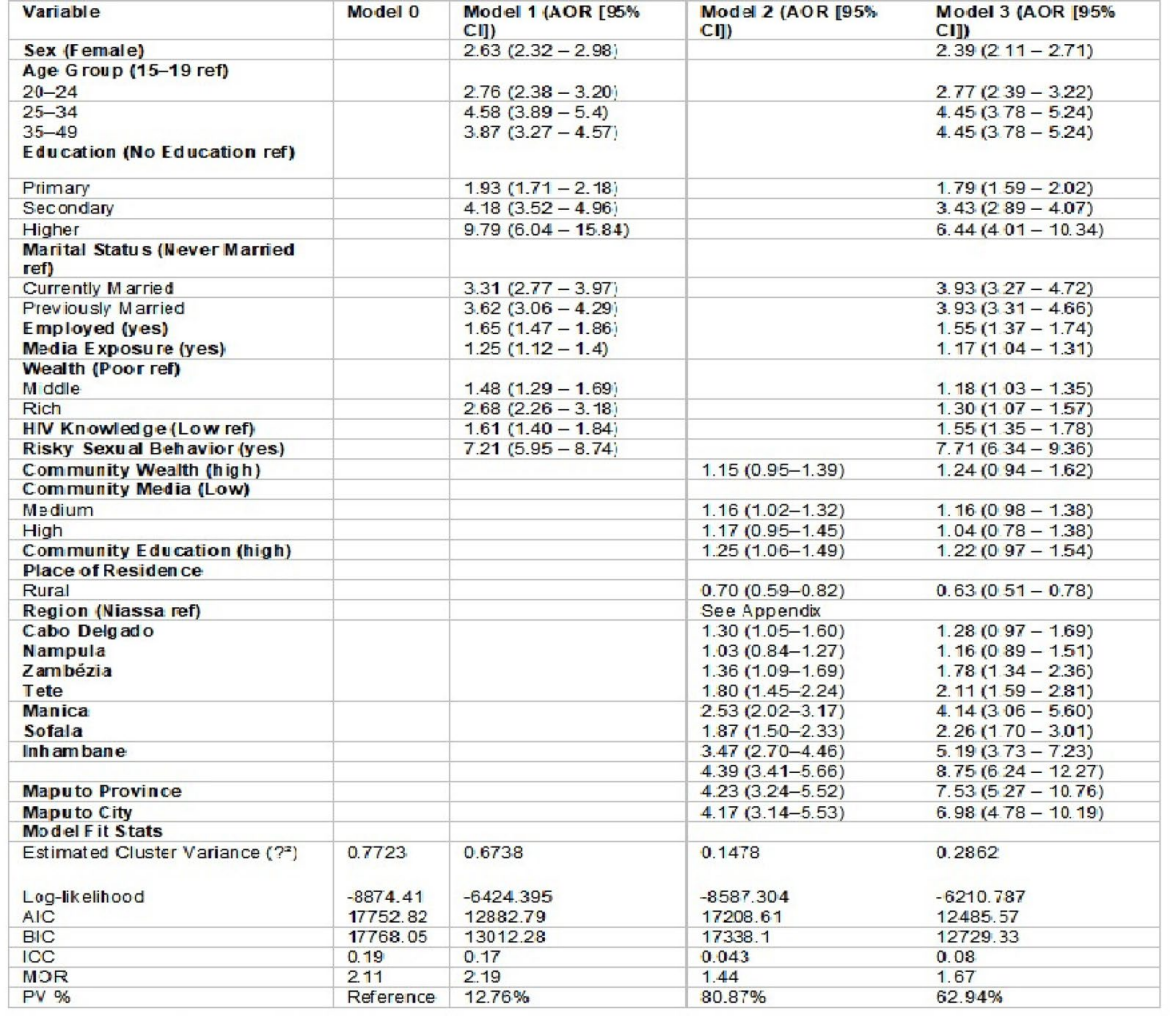
Multilevel Logistic Regression of Factors Associated with HIV Testing Uptake Among High-Risk Adults in Mozambique, 2022–2023 MDHS

## Discussion

The findings reveal that lifetime HIV testing uptake among high-risk adults in Mozambique remains suboptimal, with 63.7% (95% CI: 62.9%–64.5%) having ever tested for HIV. Significant regional disparities exist, with the highest testing rates in Cidade de Maputo (87.7%), Maputo (86.3%), and Gaza (84.2%), while provinces like Inhambane (21.3%) and Manica (25.2%) report much lower uptake.

This study shows substantial spatial variation in HIV testing uptake across Mozambique, with a pronounced cluster of low uptake in the Northern provinces. The primary cluster spanning Niassa, Cabo Delgado, Nampula, and northern Zambézia reflects persistent structural and contextual barriers, including sparse health facilities, poor transportation networks, and ongoing insecurity in Cabo Delgado. These factors collectively limit service delivery and access to HIV testing. Similar challenges in northern Mozambique have been highlighted in previous studies documenting how conflict, remoteness, and weak infrastructure hinder HIV prevention and treatment services [44, 45].

Lower educational attainment, widespread poverty, and limited exposure to health information further contribute to reduced testing uptake in these areas, mirroring well-established associations between socioeconomic disadvantage and low HIV service utilization [46].

A smaller but statistically significant secondary cluster detected in central Mozambique indicates additional localized gaps, particularly in parts of Sofala and Manica. These areas may experience health system inefficiencies, climate-related disruptions such as cyclones, or sociocultural norms that influence engagement with HIV prevention services, patterns consistent with previous analyses showing how environmental shocks and cultural dynamics shape health-seeking behaviors [47, 48]. The presence of this micro-cluster highlights the value of spatial analytical methods such as SaTScan in identifying localized public health vulnerabilities that may not be visible in aggregated national data [5, 49].

In contrast, southern provinces such as Maputo, Gaza, and Inhambane showed predominantly low-risk clusters, indicating relatively high HIV testing uptake. These regions benefit from stronger health infrastructure, greater urbanization, and higher literacy, which are known to facilitate greater uptake of HIV services in sub-Saharan Africa [50, 51]. The clear south–north gradient observed in this study aligns with prior evidence demonstrating persistent geographic disparities in health resource distribution and service utilization across Mozambique [52].

Urban residents demonstrate considerably higher testing coverage (76.2%) compared to rural populations (56.3%), indicating persistent inequities in access to HIV services. These geographic and residential disparities align with findings from other sub-Saharan African settings, where urbanization and proximity to health facilities are key facilitators of HIV testing uptake [50, 53]. Although this study assessed lifetime HIV testing uptake (prevalence), which provides a cumulative measure of service coverage and awareness over time, it does not capture recent or repeat testing behaviors. The DHS dataset used lacks information on the timing of the most recent HIV test, limiting our ability to assess testing incidence or trends. Nonetheless, lifetime testing prevalence remains an important public health indicator, as it reflects the extent to which individuals have ever accessed HIV testing services, a critical first step toward diagnosis and linkage to care. Future surveys that include time-bound testing questions could help evaluate program effectiveness in promoting regular testing among high-risk populations.

Multilevel regression analysis further identified important individual and community-level determinants. Females were more than twice as likely to have been tested as males (AOR: 2.39; 95% CI: 2.11–2.71), consistent with evidence that women often access health services more frequently, partly due to antenatal care opportunities [54, 55]. Female respondents were more likely to test for HIV, likely due to greater health service contact during antenatal care [56]. This likely reflects targeted HIV testing campaigns aimed at women, especially during antenatal care, and possibly greater health-seeking behavior among women.

Adults aged 25–34 showed the highest odds (AOR ~ 4.5) of lifetime HIV testing compared to adolescents aged 15–19. The elevated testing in this group may be due to greater sexual activity, increased risk perception, and more frequent interaction with health services. Interestingly, even older adults [35–49] maintained similarly high odds [57].

Educational attainment showed a strong positive association, with secondary (AOR: 3.43; 95% CI: 2.89–4.07) and higher education (AOR: 6.44; 95% CI: 4.01–10.34) greatly increasing the likelihood of testing, highlighting the role of knowledge and empowerment in health decisions [58]. Education likely improves awareness of HIV, reduces stigma, and increases health literacy, which encourages testing [59, 60]. Educational attainment often correlates with socioeconomic status and access to health information [61].

Being currently married was also linked to increased testing (AOR: 3.93; 95% CI: 3.27–4.72), possibly due to marriage often entailing increased sexual activity and childbearing, which can motivate testing, especially where couple or antenatal testing programs exist [62]. Similar findings were reported in studies from Ethiopia, Kenya, and Malawi, where married individuals demonstrated significantly higher testing rates than their never-married counterparts [63, 64]. Employment and wealth were significant determinants of lifetime HIV testing uptake in this study. Employed individuals had about 1.55 times higher odds of ever testing (AOR = 1.55; 95% CI: 1.37–1.74), which may reflect improved financial capacity, better health literacy, and greater exposure to workplace health programs that promote HIV testing [65, 66]. Employment often correlates with increased social networks and access to information, which can facilitate health-seeking behaviors [13]. Employment in South Africa has been shown to enhance access to HIV-related services through economic and informational pathways, according to a study by Maugham-Brown [67]. Regarding wealth, participants in the middle wealth category had modestly higher odds of testing compared to the poorest group (AOR = 1.18; 95% CI: 1.03–1.35), and those in the richest category had an even stronger association (AOR = 1.30; 95% CI: 1.07–1.57). This gradient suggests that economic resources enable individuals to overcome structural barriers such as transportation costs, clinic fees, or opportunity costs of attending health services [65, 68]. Wealthier individuals may also have better access to media and educational resources that encourage HIV testing [66]. These findings are consistent with broader literature highlighting socioeconomic status as a critical factor influencing HIV service utilization. For instance [65], found that wealthier women across sub-Saharan Africa had significantly higher HIV testing uptake, driven by enhanced access and empowerment [13]. Similarly, emphasized how poverty limits health service engagement, including HIV testing, due to both economic and social constraints. Media exposure contributed modestly but significantly to lifetime HIV testing uptake, with individuals exposed to media having 1.17 times higher odds of testing compared to those without such exposure (AOR = 1.17; 95% CI: 1.04–1.31). This association is consistent with previous studies in sub-Saharan Africa, which have shown that exposure to HIV-related information through radio, television, and print media increases awareness, improves HIV knowledge, and reduces misconceptions, thereby [13, 69, 70]. Mass media campaigns have been recognized as a cost-effective public health strategy, particularly in resource-limited settings, because they reach large populations and can be tailored to address cultural beliefs and stigma [65]. Studies show that regular exposure to HIV prevention messages via media increases HIV testing rates among young women in Zimbabwe and Rwanda, emphasizing the need for culturally sensitive messages in media to expand testing coverage [71, 72].

Importantly, individuals reporting risky sexual behavior had substantially higher odds of HIV testing. This suggests awareness of risk and motivation to seek testing among high-risk groups, which is critical for targeted interventions [60]. At the community level, wealth, education, and media exposure had limited independent effects after controlling for individual factors, indicating that individual characteristics dominate. However, residence in rural areas was associated with lower testing odds, consistent with known barriers such as limited access to health facilities and services [68]. Increased HIV-related knowledge was a strong predictor of testing uptake, which is well-documented in the literature. Knowledge about transmission, prevention, and the benefits of early diagnosis encourages individuals to seek testing voluntarily. A multi-country analysis by [16] confirmed that HIV-related knowledge significantly correlates with voluntary HIV testing, particularly among youth and high-risk populations.

There were significant regional disparities in HIV testing uptake. Provinces such as Inhambane and Manica showed substantially higher odds compared to the reference region. These differences may reflect variations in health infrastructure, availability of testing services, and cultural acceptance of HIV testing [73, 74].

Overall, the model explains a considerable proportion of variability, with both individual and contextual factors playing roles, although individual-level determinants appear more influential. These findings underscore the importance of multi-level approaches that address both personal and structural barriers to HIV testing [9, 13]. The current findings are in line with existing literature and reinforce the importance of socio-demographic and behavioral factors in shaping HIV testing behaviors in high-burden settings like Mozambique. Model diagnostics indicated that the full model (Model 3) provided the best fit (AIC = 12485.57; BIC = 12729.33), with the intraclass correlation coefficient decreasing from 0.19 in the null model to 0.08 and a proportional change in variance of 62.94%. This substantial reduction in unexplained cluster-level variation confirms the relevance of multilevel modeling in capturing contextual disparities. The findings emphasize the pivotal role of socio-demographic, informational, and regional determinants in shaping HIV testing uptake, underscoring the need for targeted, equity-driven interventions, particularly for adolescents, men, and rural populations.

### Strengths and limitations

This study used nationally representative 2022–2023 MDHS data, ensuring generalizability to Mozambique’s adult population. A large sample and multilevel logistic regression allowed examinations of individual- and community-level determinants while accounting for clustering. Standardized DHS procedures enhanced data reliability and comparability, and the use of spatial techniques hotspot mapping and SaTScan—provided valuable geographic insights into HIV testing distributions.

Limitations include the lack of data on the timing of the most recent HIV test, restricting analysis to lifetime (ever-tested) uptake rather than recent or repeat testing. The cross-sectional design limits causal inference, and self-reported testing may be affected by recall or social desirability bias. Residual confounding from unmeasured factors such as stigma, accessibility, and service quality is possible. Community-level measures may not perfectly capture actual characteristics, and while spatial analyses identified geographic patterns, they do not explain causal mechanisms. Although Bayesian CAR models could better adjust for spatial autocorrelation, this study relied on Kriging and SaTScan for feasibility and interpretability, still providing meaningful insights into geographic variation in HIV testing. We did not perform a separate sensitivity analysis excluding respondents with missing data. However, the use of survey-weighted analyses minimizes potential bias from missing observations.

## Conclusion

Despite moderate overall uptake, more than one-third of high-risk adults in Mozambique remain untested for HIV, indicating a substantial coverage gap. Testing uptake is strongly influenced by sex, age, education, and risky sexual behavior, with rural residence significantly reducing the likelihood of testing. Spatial analyses revealed clear geographic inequalities, with persistent low-uptake clusters in Niassa, Zambézia, and parts of Nampula, and high-uptake areas in Maputo Province and Inhambane.

### Policy implication

HIV testing programs should adopt targeted, geographically focused strategies that intensify outreach in low-uptake regions, integrate services for men and rural communities, and leverage education and media interventions to close the testing gaps.

## Data Availability

The DHS datasets are available from the DHS Program ([https://dhsprogram.com](https:/dhsprogram.com)) upon reasonable request and approval. Direct link to the dataset listing: [https://dhsprogram.com/data/available-datasets.cfm](https:/dhsprogram.com/data/available-datasets.cfm). All GPS data were displaced as per DHS guidelines. No sensitive location information is reported, and cluster coordinates are presented at an aggregated level to maintain confidentiality.
